# Comparative Immune Response in Children and Adults with *H. pylori* Infection

**DOI:** 10.1155/2015/315957

**Published:** 2015-10-01

**Authors:** Alireza Razavi, Nader Bagheri, Fatemeh Azadegan-Dehkordi, Mahsa Shirzad, Ghorbanali Rahimian, Mahmoud Rafieian-Kopaei, Hedaytollah Shirzad

**Affiliations:** ^1^Department of Immunology, School of Public Health, Tehran University of Medical Sciences, Tehran, Iran; ^2^Cellular and Molecular Research Center, Shahrekord University of Medical Sciences, Shahrekord, Iran; ^3^School of Dentistry, Isfahan University of Medical Sciences, Isfahan, Iran; ^4^Department of Internal Medicine, Shahrekord University of Medical Sciences, Shahrekord, Iran; ^5^Medical Plants Research Center, Shahrekord University of Medical Sciences, Shahrekord, Iran

## Abstract

*Helicobacter pylori* (*H. pylori*) infection is generally acquired during early childhood; therefore, the immune response which usually takes place at this age may influence or even determine susceptibility to the infection contributing to the clinical outcomes in adulthood. Several cytokines including IL-6, IL-10, and TGF-*β*1 as well as Foxp3^+^ cell numbers have been shown to be higher; however, some other cytokines consisting of IL-1*β*, IL-17A, and IL-23 are lower in infected children than in infected adults. Immune response to *H. pylori* infection in children is predominant Treg instead of Th17 cell response. These results indicate that immune system responses probably play a role in persistent *H. pylori* infection. Childhood *H. pylori* infection is also associated with significantly lower levels of inflammation and ulceration compared with adults. This review, therefore, aimed to provide critical findings of the available literature about comparative immune system in children and adults with *H. pylori* infection.

## 1. Introduction

*Helicobacter pylori* (*H. pylori*) infections usually occur during childhood, continue throughout the life, and cause severe diseases such as gastritis, gastric ulcer, gastric carcinoma, and duodenum ulcer in adulthood.* H. pylori* is a well-known gastric pathogen infecting more than half of the world's people [1]. The outcomes of* H. pylori* infection seem to be dependent on some factors like gene regulation factors, genetic predisposition of the patient, receptor gene polymorphisms, particular cytokine, constituents, and environmental influences [2, 3]. Fortunately, most of the infected children do not develop any complications; however, the immunological events which usually develop in the children gastric mucosa are probably decisive in the immune response determining the final outcome the infection. The colonization of the stomach by this pathogen bacterium causes an inflammatory response and recruits neutrophils, lymphocytes, dendritic cells, and macrophages, to the gastric mucosa [4, 5]. There are complex mechanisms by which* H. pylori* may start and maintain the local immune response; however, cytokines produced by both adaptive immune and innate systems may lead to the development of gastric mucosa-associated lymphoid tissue lymphoma, gastric adenocarcinoma, and other ulcerative diseases; studies in* H. pylori* infection have revealed that childhood* H. pylori* infection is usually associated with significantly lower levels of gastric inflammation and ulceration in comparison to adults. Therefore, this review study was aimed to provide the critical findings of the available literature about comparative immune system in children and adults with* H. pylori* infection.

## 2. Bacterial Virulence Factors

*Helicobacter pylori* may express the virulence factors associated with inflammation as well as inflammatory symptoms in infected patients. The main pathogenicity factors of* Helicobacter pylori* include *γ*-glutamyl transpeptidase (GGT), cytotoxin-associated gene A (cagA) product, and virulence components vacuolating toxin (vacA), in addition to pathogen-associated molecular patterns (PAMPs) such as flagella and lipopolysaccharide (LPS) [6–9]. The cytotoxin-associated gene (cag) pathogenicity island (PAI) is one of these factors which has been extensively studied in regard to inflammation [10–12]. Colonization with the strains that possess cagA is more frequently associated with peptic ulceration gastric adenocarcinoma or other gastric mucosal complications than the cagA strains [13, 14]. It has been shown that cagA may play a role in production of IL-8 as well as activation of nuclear factor kappa-B (NF-*κ*B) [15]. Furthermore, expression of cagA induces production of IL-8 and translocation of NF-*κ*B nuclear in gastric epithelial cells [13, 16]. The vacA from* H. pylori* is capable of inducing intracellular vacuolation in gastric epithelial cells. Hence, it has been hypothesized that it may contribute in damage of gastric and duodenal mucosa which ultimately leads to ulcer formation,* in vivo* [14]. Moreover, the bacterial virulence factors vacA and cagA have important roles in pathogenesis of* H. pylori* infection. Others like blood group antigen-binding adhesion (BabA), outer inflammatory protein (oipA), sialic acid-binding adhesion (sabA), iceA (induced by contact with epithelium), and duodenal ulcer promoting gene (dupA) may promote colonization of the mucosa, too [17]. In regard to virulence factors cagA and vacA, these bacteria are very heterogeneous [18]. A lot of evidences have revealed that these genetic variations may have an important role in the outcome of infection [19, 20].

## 3. T Cell Subsets

T helper (Th) cells have been shown to differentiate into functional classes of two major CD4^+^ including Th1 cells (able to produce some cytokines such as IL-2 and IFN-*γ*) and Th2 cells (producing cytokines like IL-4, IL-5, and IL-10) [21, 22]. Th1 cells mediated cell immunity, which has an important role against intracellular parasites. However, Th2 generates humoral immunity as well as prevention of intestinal helminthes [23]. Other than Th1/Th2 paradigm, a unique subset of IL-17 producing Th17 cells has been discovered [24–26]. IL-23 has a crucial role in differentiation of Th17 cells. However, IL-4 and IL-12 promote, respectively, Th1 and Th2 cell differentiation [27]. It has been revealed that IL-17 possesses 6 family members (IL-17A–F), IL-17A (simply called IL-17) being the prototypic IL-17 family member [28, 29]. Furthermore, IL-17A exerts proinflammatory effects by stimulation of the production of chemokines such as IL-1, IL-6, cytokines, monocyte chemoattractant protein-1, and upregulation of cell adhesion molecules like vascular cell adhesion molecule-1 and intercellular adhesion molecule-1. The IL-17A plays a crucial role in induction of autoimmune diseases such as inflammatory bowel disease (IBD), experimental autoimmune encephalomyelitis (EAE), and rheumatoid arthritis (RA), as well as chronic inflammatory diseases [30–33]. Regulatory T (Treg) cells, by proliferation of antigen specific T cells and suppressing the activation, have important role in chronic inflammation. It should be noted that depletion or dysfunction of Treg cells is usually associated with inflammatory bowel disease, allergy, and autoimmune disease [34]. Treg cells comprise different subsets: Tr1 cells secreting interleukin IL-10, Th3 cells characterized by transforming growth factor (TGF-*β*1) secretion, and naturally occurring FOXP3-expressing CD4^+^CD25^high^ Treg cells [35, 36]. The FOXP3^+^CD4^+^CD25^high^ Treg cells are further divided into two subsets: thymus derived naturally occurring FOXP3^+^CD4^+^CD25^high^ Treg cells and peripherally induced FOXP3^+^CD4^+^CD25^high^ Treg cells [37].

## 4. Differences in Immunity of Children and Adults Infected and Uninfected with* H. pylori*

**Figure 1:**
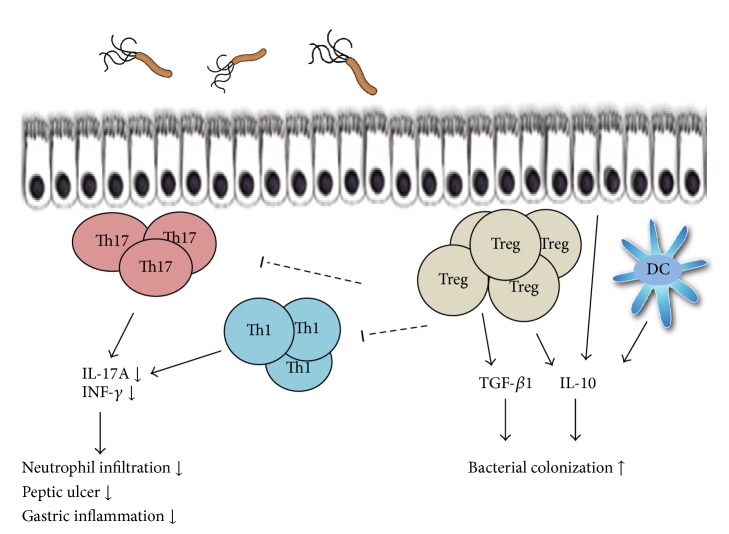
Diagram of how the Treg cell response may influence inflammation, bacterial colonization density, and occurrence of* H. pylori*-mediated disease.

The human gastric mucosal biopsies revealed that people who were persistently infected with* H. pylori*, in comparison to uninfected ones, show an increased and higher level of infiltrated various types of leukocytes [38]. In these specimens, lymphocytes (T and B cells), monocytes, mast cells, neutrophils, macrophages, eosinophil, and dendritic cells are usually present [2, 4]. CD4^+^ T cells, B cells and dendritic cells may be organized in lymphoid follicles [39] indicating ongoing chronic immune responses and antigen presentation. In peripheral blood and gastric mucosa of infected humans, the* H. pylori*-specific CD4^+^ T cells are detectable which is not detectable in uninfected individuals [40]. The cytokines such as TNF-*α*, IFN-*γ*, IL-1, IL-6, IL-7, IL-8, IL-10, IL-17, IL-18, and IL-23 have usually increased levels in the stomach of* H. pylori*-infected patients in comparison to the uninfected subjects [2, 41, 42]. IL-4 is not usually detectable in the gastric mucosa of* H. pylori*-infected patients [43]. Hence, to show, in children, the mucosal regulation of* H. pylori* infection, which can provide a window to the early host response to bacteria, the mucosal cytokine response to the infection, the associated cellular infiltrate, and the characterized bacteria might be helpful. Studies on* H. pylori*-infected children and adults have shown that children possess reduced gastric inflammation in comparison to the infected adults, in spite of similarity in* H. pylori* colonization level. Furthermore, inflammation in children has been shown to be less in comparison to that of adults, indicating a downregulation in immune response to infection in children [44, 45]. Moreover, the sequence analysis revealed that the bacteria isolated from the infected children and adults might have similar* cagA* and* vacA* gene profiles. The difference in bacterial strains and common virulence factors were not the cause of low level of inflammation in infected children in comparison to adults [44].* H. pylori*-infected children in comparison to infected adults possess lower levels of protein and gastric IL-17-specific mRNA as well as fewer gastric Th17 cells, indicating more reduction in the mucosal Th17 response in infected children. Moreover, the gastric mucosa of the infected children has lower level of IFN-*γ* mRNA, confirming the findings indicated of reduced Th1 response in children with* H. pylori* infection [46, 47]. Recent study indicated that the gastric concentrations of cytokines representative of the innate and Th1 response were higher in the* H. pylori*-positive than in the* H. pylori*-negative children and adults. The gastric concentrations of IL-1*α* and TNF-*α* were significantly higher, while those of IL-2, IL-12p70, and IFN-*γ* were lower in the infected children than in the infected adults. In the infected children, the gastric concentration of IL-1*α*, IL-2, IL-12p70, and IFN-*γ* increased, whereas in adults the gastric concentrations of IFN-*γ* and IL-12p70 decreased with aging. Increased gastric concentration of Th1-associated cytokines correlated with increased degree of gastritis, that is, the background lesion for the development of the* H. pylori*-associated severe diseases [48]. Treg cells are described as the key regulator of the immune system in the maintenance of immunologic tolerance. Recently, the close relationship between* H. pylori* infection and immunosuppressive Treg cells has been reported in animal and human models [49]. Treg cells suppress* H. pylori*-induced Th1-mediated immune response to contribute to the bacteria's persistent colonization in the gastric mucosa and therefore may play a major role in inducing chronic gastritis. The TGF-*β*1 and IL-10 gastric levels and the gastric number of Treg Foxp3^+^ cells in* H. pylori*-positive groups are higher in children than in adults (Table 1) [44, 46, 49–51]. The consensus is that Treg cells and Th17 commitments might be mutually controlled. TGF-*β* is required for the differentiation of both Treg cells and Th17 by inducing key transcription factors, Foxp3 and ROR*γ*t/RORc, respectively [52–54]. But, in absence of IL-6, an exclusive Treg differentiation might occur as Foxp3 is capable of associating with and inhibiting the ROR*γ*t. In contrast, in presence of IL-6, this inhibition might be abrogated allowing Th17 differentiation [55]. In a paradoxical pattern, the gastric concentration of IL-6 is usually less in infected adults than in infected children. In this regard, it might be hypothesized that these results are due to the higher gastric levels of IL-23 in adults, compared with children, which might prevent the amplification/stabilization of the shifted Th17 cells. The other possibility might be the higher level of TGF-*β* in the gastric milieu of infected children. It should be noted that, at low levels, TGF-*β* synergizes with IL-6 to promote IL-23 receptor expression in favor of Th17 cell commitment. However, the high level of TGF-*β* represses IL-23 receptor expression favoring Foxp3^+^ Treg cell differentiation [49, 56]. A recently published study revealed that IL-6 overproductions by IL-6 transgenic mice do not affect the function and development of natural Treg [57]. In this regard, the predominant Treg differentiation in children infected with* H. pylori* might account for more susceptibility of children to the* H. pylori* infection as well as to the bacterium persistence. Study in mouse stomach showed that* H. pylori*-induced dendritic cells skew the Th17/Treg balance toward a Treg-biased response that suppresses Th17 immunity through a cagA and vacA independent, TGF-*β* and IL-10-dependent mechanism [58, 59]. In support of these findings, recent study showed that* H. pylori* was capable of stimulating human gastric dendritic cells to produce IL-10, potentially supplementing Treg suppression of inflammation in the gastric mucosa [60, 61] (Figure 1). From these findings we might conclude that* H. pylori*-induced gastritis in adult is the consequence of both Th1 and Th17 immune-mediated inflammatory pathway involvement and that both pathways might be downregulated in the gastric mucosa of infected children.

## 5. Conclusion

In conclusion,* H. pylori* infection in children is associated with high Treg response, as well as low Th1 and Th17 response [44, 46]. But,* H. pylori*-specific Th17/Th1 detection in chronically infected patients may reveal that the initial response is progressively lost [43, 62], indicating that, with progression of time, the mucosal immune system probably identifies* H. pylori*, as a pathogen. Hence, Th1, Th17, and Treg results may imply gastric mucosal response to* H. pylori*. More data from immune-mediated mechanism(s) of mucosal inflammation is required to provide strategies against this challenging pathogen, particularly for children who are living in countries with high rate of gastric cancer and/or* H. pylori* infection.

## Figures and Tables

**Table 1:** Comparative immune response and clinical outcome in children and adults infected with *H. pylori*.

	Children	Adults
Th1	↓	↑
Th17	↓	↑
Treg	↑	↓
TGF-*β*1	↑	↓
IL-10	↑	↓
Gastric inflammation	↓	↑
Neutrophil infiltration	↓	↑
Peptic ulcer	↓	↑
Virulence factors	Similar	Similar
